# Sorafenib for the treatment of advanced hepatocellular carcinoma with extrahepatic metastasis: a prospective multicenter cohort study

**DOI:** 10.1002/cam4.548

**Published:** 2015-10-16

**Authors:** Masahito Nakano, Masatoshi Tanaka, Ryoko Kuromatsu, Hiroaki Nagamatsu, Nobuyoshi Tajiri, Manabu Satani, Takashi Niizeki, Hajime Aino, Shusuke Okamura, Hideki Iwamoto, Shigeo Shimose, Tomotake Shirono, Hironori Koga, Takuji Torimura, the Kurume Liver Cancer Study Group of Japan

**Affiliations:** ^1^ Division of GastroenterologyDepartment of MedicineKurume University School of MedicineKurumeFukuokaJapan; ^2^ Yokokura HospitalMiyamaFukuokaJapan; ^3^ Yame General HospitalYameFukuokaJapan

**Keywords:** Extrahepatic metastasis, hepatic reserve, hepatocellular carcinoma, long‐term treatment, sorafenib

## Abstract

Sorafenib, an oral multikinase inhibitor, is approved for advanced hepatocellular carcinoma (HCC) treatment. However, its therapeutic effect in advanced HCC patients with extrahepatic metastasis remains uncertain. This study aimed to prospectively assess the efficacy, safety, and survival risk factors and evaluate the prognostic impact of sorafenib treatment in advanced HCC patients with or without extrahepatic metastasis. Between May 2009 and March 2014, 312 consecutive advanced HCC patients who received sorafenib were enrolled in this study. We evaluated their characteristics and compared the clinical outcomes of those with and without extrahepatic metastasis. Of the enrolled patients, 245 (81%) received sorafenib treatment for more than 1 month, with a median duration of 3.6 months. Eighteen patients demonstrated partial response to sorafenib therapy, 127 had stable disease, and 134 had progressive disease at the first radiologic assessment. The median survival time (MST) and progression‐free survival (PFS) were 10.3 and 3.6 months, respectively. Multivariate analysis identified gender, Child‐Pugh class, baseline serum des‐gamma‐carboxy prothrombin level, and treatment duration as independent risk factors for survival. Extrahepatic metastasis was detected in 178 patients. However, the MST, PFS, and therapeutic effect were comparable between patients with and without extrahepatic metastasis. The independent risk factors for decreased overall survival in patients with extrahepatic metastasis were similar to those affecting all patients. Our results indicated that sorafenib could be administered for hepatic reserve and as long‐term treatment for advanced HCC patients regardless of their extrahepatic metastasis status.

## Introduction

Hepatocellular carcinoma (HCC) is one of the most common malignancies in the world 1. Recent advances in imaging have increased the early detection rate of HCC. Curative therapies, such as hepatic resection, liver transplantation, and radiofrequency ablation, are possible in early‐stage HCC and thus improve patient survival rates 2, 3. Otherwise, transarterial chemoembolization is an important locoregional treatment for patients with unresectable HCC 4. However, long‐term survival remains limited due to high rates of recurrence, even after such curative therapies 5. In particular, the development of advanced HCC with macroscopic vascular invasion or extrahepatic metastasis significantly reduces survival rates, as no effective systemic therapies are available to date 6, 7.

Recently, sorafenib, an oral multikinase inhibitor, has been approved as a new molecularly targeted therapy for advanced HCC. The magnitude of benefit obtained with sorafenib is similar to that with trastuzumab in breast cancer, bevacizumab in colon cancer, or erlotinib in lung cancer 8, 9, 10. Sorafenib has been shown to suppress tumor growth and angiogenesis by inhibiting the Raf/MEK/ERK signaling pathway and receptor tyrosine kinases, such as vascular endothelial growth factor receptor (VEGFR) 1, VEGFR‐2, VEGFR‐3, and platelet‐derived growth factor receptor beta 11.

The introduction of sorafenib has changed the standard systemic therapy for advanced HCC, as demonstrated by recent positive results from randomized controlled trials, and this new treatment was approved in Japan in May 2009 12, 13, 14. The strong evidence of sorafenib offering significant survival benefit in advanced HCC was derived from the SHARP (Sorafenib HCC Assessment Randomized Protocol) trial 12. Another phase III clinical trial enrolling patients from the Asia‐Pacific region concluded that sorafenib was a well‐tolerated and effective treatment for advanced HCC 15.

Extrahepatic metastasis of HCC remains the leading cause of death from the disease 16. To date, the prognostic factors for patients with extrahepatic metastasis remain unclear. Previous studies reported that these patients had a worse prognosis than those without extrahepatic metastasis 17. The lungs are the most common organ for extrahepatic spread, but most pulmonary metastases are multifocal and often unsuitable for surgical resection 17. Thus, the clinical outcome and prognosis of patients with extrahepatic metastasis treated with sorafenib require further investigation.

Therefore, in the present study, we prospectively assessed the efficacy and safety of sorafenib, identified the factors associated with improved survival in advanced HCC patients, and evaluated the prognostic impact of extrahepatic metastasis status.

## Materials and Methods

### Patients

Eligibility criteria for this study were similar to those of the SHARP trial. Briefly, all enrolled patients met the following requirements: (1) Eastern Cooperative Oncology Group performance status of 0–1, (2) measurable disease using the Response Evaluation Criteria in Solid Tumors (RECIST) 18, (3) Child‐Pugh class A or B liver function, (4) leukocyte count of ≥2000/mm^3^, (5) platelet count of ≥50 × 10^9^/L, (6) hemoglobin level of ≥8.5 g/dL, (7) serum creatinine level of <1.5 mg/dL, and (8) no ascites or encephalopathy. Between May 2009 and March 2014, 312 patients diagnosed with advanced HCC were enrolled in this study. HCC was either confirmed via histological studies or diagnosed using noninvasive criteria according to the European Association for the Study of Liver 19. Enrolled patients were treated with sorafenib at one of the 13 experienced member institutions of the Kurume Liver Cancer Study Group of Japan: Asakura Medical Association Hospital, Chikugo City Hospital, Kurume General Hospital, Kurume University Medical Center, Kurume University School of Medicine, Kyushu Medical Center, O–muta City Hospital, Saga Social Insurance Hospital, Social Insurance Tagawa Hospital, St. Mary's Hospital, Tobata Kyouritsu Hospital, Yame General Hospital, and Yokokura Hospital. The primary outcome of this study was overall survival time, which was defined as the time from sorafenib treatment initiation to the date of death or the patient's last follow‐up. Relevant data from all patients’ clinical records, including medical history, laboratory results, radiologic findings, histologic results, and survival data, as well as the dosage and adverse events associated with sorafenib therapy, were prospectively collected. The study protocol was approved by the Ethics Committee of Kurume University (No. 10009) and University Hospital Medical Information Network (UMIN) Center (No. UMIN000007427), and conformed to the guidelines of the 1975 Declaration of Helsinki. Patients were given comprehensive information on the details of the clinical study, and they provided written informed consent prior to participation.

### Diagnosis

Intrahepatic lesions and vascular invasion were diagnosed using a combination of contrast‐enhanced computed tomography (CT), magnetic resonance imaging (MRI), ultrasonography (US), and digital subtraction angiography. In addition, alpha‐fetoprotein (AFP), lens culinaris agglutinin‐reactive fraction of AFP (AFP‐L3), and des‐gamma‐carboxy prothrombin (DCP) serum levels were measured up to 1 month prior to treatment. Intra‐abdominal metastases were detected via abdominal CT, MRI, and US, which were performed to evaluate intrahepatic lesions. Pulmonary lesions were detected on chest radiography or chest CT, which was routinely performed up to 1 month prior to treatment. Additional examinations, such as bone scintigraphy and brain CT or MRI, were indicated when symptoms attributable to extrahepatic metastasis appeared. These examinations were also conducted when AFP, AFP‐L3, or DCP levels were elevated, and the elevation could not be explained by the status of the intrahepatic lesions 19. Tumor stage was determined according to the Barcelona Clinic Liver Cancer (BCLC) staging classification 20.

### Sorafenib treatment

Performance status was used to determine the initial sorafenib dosage as per the chief physician's discretion. Discontinuation and dose reduction were allowed based on tolerance. Side effects of sorafenib treatment were documented according to the National Cancer Institute's Common Terminology Criteria for Adverse Events (CTCAE), version 4.0. Treatments were discontinued upon development of CTCAE grade 3 or higher adverse events with the exception of a platelet count of <25 × 10^9^/L and a leukocyte count of <1500/mm^3^.

### Assessment of tumor response

Imaging studies were performed 4 weeks after the initiation of sorafenib treatment and every 4–6 weeks thereafter to assess tumor response. The assessment was conducted according to the RECIST, version 1.1 18 as follows: complete response (CR), all measurable lesions disappeared for more than 4 weeks; partial response (PR), the sum of the diameters of the largest target lesions decreased by more than 30%, and there was no development of a new lesion for more than 4 weeks; progressive disease (PD), the sum of the largest diameters increased by more than 20%, or a new lesion appeared; and stable disease (SD), neither PR nor PD was observed 21. Patients who died before their first radiographic assessment were classified as having PD. The time to radiologic progression was defined as the time from sorafenib treatment initiation to disease progression. Data from patients who died without tumor progression were censored. The disease control rate was defined, on the basis of independent radiologic review, as the percentage of patients whose best‐response RECIST rating of CR, PR, or SD was maintained for at least 1 month after the first demonstration of such rating.

### Statistical analysis

Baseline patient characteristics were analyzed using descriptive statistical methods. Survival curves were calculated using the Kaplan–Meier method. Univariate analysis of survival curves was performed using the log‐rank test. A *P*‐value of <0.05 was considered statistically significant. The Cox proportional hazards model was used to evaluate the interaction between baseline characteristics and the effect of sorafenib on overall survival. JMP software (SAS Institute, Inc., Cary, NC), version 11, was used for all analyses.

## Results

### Patient characteristics

The study cohort included 241 (77%) men and 71 (23%) women, with a mean age of 72 years (Table 1). Chronic hepatitis C virus infection was the predominant cause of HCC (*n* = 189; 62%), followed by chronic hepatitis B virus infection (*n* = 55; 23%). Of the enrolled patients, 165 (53%) had a Child‐Pugh score of 5 and 100 (32%) had a Child‐Pugh score of 6. Overall, 265 (85%) patients had Child‐Pugh class A, whereas 47 (15%) had class B liver cirrhosis. According to the BCLC staging system, 100 (32%) patients had stage B disease, and 212 (68%) had stage C. Prior to sorafenib therapy, 277 (89%) patients had been treated with surgical, locoregional, or pharmacologic therapies. Of these, 176 received transcatheter arterial chemoembolization, 107 were treated with hepatic arterial infusion chemotherapy, 92 underwent hepatic resection, and 76 had radiofrequency ablation.

**Table 1:** Characteristics of the total cohort (no. with % and median with range)

Variable	
Age, years [range]	72.0 [33–94]
Gender, *n* (%)	
Male	241 (77.2)
Female	71 (22.8)
Etiology, *n* (%)	
HBV	55 (17.6)
HCV	189 (60.6)
Both negative	68 (21.8)
Child‐Pugh class, *n* (%)	
A	265 (84.9)
B	100 (32.1)
BCLC stage, *n* (%)	
B	100 (21.2)
C	212 (84.9)
Initial sorafenib dose, *n* (%)	
400 mg	209 (67.0)
800 mg	103 (33.0)
Extrahepatic metastasis, *n* (%)	178 (57.0)
Lung	105 (33.7)
Bone	40 (12.8)
Lymph node	38 (12.2)
Peritoneum	17 (5.4)
Adrenal gland	11 (3.5)
Macrovascular invasion, *n* (%)	
Presence	81 (26.0)
Absence	231 (74.0)
Albumin—median in g/L [range]	3.50 [2.39–4.70]
Total bilirubin—median in mg/dL [range]	0.78 [0.15–3.70]
Prothrombin time—median in % [range]	83.3 [10.8–136.0]
AFP—median in ng/mL [range]	105 [1–987,600]
DCP—median in mAU/mL [range]	738 [2–621,000]

HBV, hepatitis B virus; HCV, hepatitis C virus; BCLC, Barcelona Clinic Liver Cancer; AFP, alpha‐fetoprotein; DCP, des‐gamma‐carboxy prothrombin.

### Treatment compliance

The initial dose of oral sorafenib administration was 400 mg daily in 209 patients and 800 mg daily in 103 patients. By the end of the follow‐up period, 273 patients had discontinued treatment. The reasons for discontinuation were adverse events in 147 patients, radiologic and symptomatic progression in 90, and deterioration of performance status in 21.

### Overall response and efficacy

The median duration of sorafenib treatment was 3.6 months (range: 0.1–49.5 months), and the median follow‐up period was 8.6 months (range: 0.4–54.3 months). Of the enrolled patients, 245 (81%) received sorafenib treatment for more than 1 month. Sixty‐seven patients who received sorafenib for less than a month were indeed treated with other therapeutic modalities, including transcatheter arterial chemoembolization, hepatic arterial infusion chemotherapy, systemic chemotherapy, or radiation therapy. In total, 228 (73%) died during the observation period and 84 (27%) were alive at the end of follow‐up. At the first radiologic assessment, 18 (6%) patients showed PR, 127 (41%) had SD, and 134 (43%) experienced PD according to the RECIST, whereas follow‐up radiologic evaluation was unavailable for 33 (10%) patients. Thus, the disease control rate was 47%.

### Factors associated with survival outcomes

**Figure 1:**
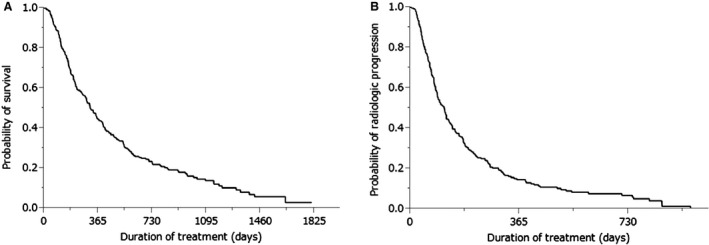
(A) Kaplan–Meier analysis of overall survival in all enrolled patients. Median survival time was 10.3 months and 1 year survival rate was 44%. (B) Kaplan–Meier analysis of radiologic progression‐free survival in all enrolled patients. Median survival time was 3.6 months.

**Figure 2:**
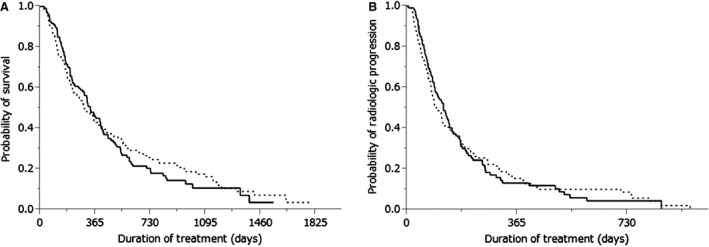
(A) Kaplan–Meier analysis of overall survival in patients with (dotted line) and without (solid line) extrahepatic metastasis (*P *=* *0.7654). Median survival time was 11.0 months versus 9.6 months, respectively. (B) Kaplan–Meier analysis of radiologic progression‐free survival in patients with (dotted line) and without (solid line) extrahepatic metastasis (*P *=* *0.8658). Median survival time was 4.0 months versus 3.2 months, respectively.

Cumulative survival curves of all patients are shown in Figures 1 and 2. The median survival time (MST) was 10.3 months (range: 0.4–54.3 months), with a 1 year survival rate of 44% (Fig. 1A). The median progression‐free survival (PFS) time was 3.6 months (range: 0.1–31.3 months; Fig. 1B).

Cox proportional hazards regression analysis was performed to identify independent factors associated with survival (Table 2). The results of univariate analysis showed that gender (men, *P *=* *0.0042), Child‐Pugh class (B, *P *=* *0.0007), serum DCP level at baseline (median level of ≥738 mAU/mL, *P *=* *0.0008), and treatment duration (median duration of ≥3.6 months, *P *<* *0.0001) were significant risk factors adversely affecting survival. Multivariate analysis confirmed that gender (men, *P *=* *0.022, hazard ratio [HR] = 0.607, 95% confidence interval [CI] = 0.406–0.930), Child‐Pugh class (B, *P *=* *0.001, HR = 2.344, 95% CI = 1.435–3.680), serum DCP level at baseline (median level of ≥738 mAU/mL, *P *=* *0.003, HR = 1.726, 95% CI = 1.203–2.495), and duration of treatment (median duration of ≥3.6 months, *P *<* *0.001, HR = 0.254, 95% CI = 0.179–0.359) were independent factors for survival.

**Table 2:** Univariate and multivariate analyses of overall survival in all patients

Variable	Univariate analysis		Multivariate analysis	
	HR (95% CI)	*P*‐value	HR (95% CI)	*P*‐value
Age (≥72 years)	1.213 (0.891–1.706)	0.2074		
Gender (male)	0.537 (0.360–0.818)	0.0042	0.634 (0.466–0.879)	0.0069
Child‐Pugh class (B)	2.419 (1.479–3.817)	0.0007	2.236 (1.529–3.189)	<0.0001
Tumor stage (BCLC‐C)	0.893 (0.634–1.277)	0.5272		
Initial dose (800 mg)	0.786 (0.547–1.112)	0.1851		
Daily average dosage (≥400 mg)	1.185 (0.821–1.717)	0.3644		
AFP (≥105 ng/mL)	1.116 (0.757–1.654)	0.5805		
AFP‐L3 (≥22%)	1.233 (0.857–1.777)	0.2596		
DCP (≥738 mAU/mL)	1.791 (1.271–2.542)	0.0008	1.728 (1.321–2.266)	<0.0001
Duration of treatment (≥3.6 months)	0.374 (0.260–0.534)	<0.0001	0.370 (0.283–0.484)	<0.0001
Therapeutic effect (PD)	1.236 (0.879–1.737)	0.2221		

HR, hazard ratio; 95% CI, 95% confidence interval; BCLC, Barcelona Clinic Liver Cancer; AFP, alpha‐fetoprotein; DCP, des‐gamma‐carboxy prothrombin; PD, progressive disease.

### Adverse events

Hand–foot skin reaction (HFSR) was the most commonly observed adverse event in our series, occurring in 145 (46%) patients. Other frequent toxicities included diarrhea (*n* = 53; 17%), fatigue (*n* = 39; 13%), liver dysfunction (*n* = 36; 12%), alopecia (*n* = 24; 8%), and hypertension (*n* = 24; 8%). The most frequent adverse event leading to discontinuation of sorafenib treatment was liver dysfunction (*n* = 43, including 35 patients with Child‐Pugh class A and 8 with class B; 14%), followed by HFSR (*n* = 12; 2%) and diarrhea (*n* = 12; 2%). Interstitial pneumonia (*n* = 2) and tumor lysis syndrome (*n* = 1) were serious adverse events. A case of interstitial pneumonia resulted in death.

### Impact of extrahepatic metastasis

Of the treated patients, 178 (57%) had extrahepatic metastasis and 134 (43%) did not. The most frequent organ sites of extrahepatic metastases were the lungs (*n* = 105), bone (*n* = 40), lymph nodes (*n* = 38), peritoneum (*n* = 17), and adrenal glands (*n* = 11). Cumulative survival curves of patients with (dotted line) and without (solid line) extrahepatic metastasis are shown in Figure 2A and B. The MST was 11.0 months for patients with extrahepatic metastasis and 9.6 months for those without (*P *=* *0.7654, Fig. 2A), whereas the corresponding PFS time was 4.0 and 3.2 months, respectively (*P *=* *0.8658, Fig. 2B). The therapeutic effect did not differ significantly between patients with and without extrahepatic metastasis (Table 3).

**Table 3:** Therapeutic effects in patients with and without extrahepatic metastasis; *P *=* *0.3061 (via chi‐square test)

Variable	With extrahepatic metastasis (*n* = 178)	Without extrahepatic metastasis (*n* = 134)
PR	8	10
SD	76	56
PD	83	56
Not evaluable	11	12

PR, partial response; SD, stable disease; PD, progressive disease.

Cox proportional hazards regression analysis was performed to identify independent risk factors associated with overall survival in patients with extrahepatic metastasis (Table 4). The results of univariate analysis showed that gender (men, *P *=* *0.0297), Child‐Pugh class (B, *P *=* *0.0182), serum DCP level at baseline (median level of ≥738 mAU/mL, *P *=* *0.0032), and duration of treatment (median duration of ≥3.6 months, *P *<* *0.0001) were significant risk factors adversely affecting overall survival in patients with extrahepatic metastasis. Multivariate analysis confirmed that gender (men, *P *=* *0.0040, HR = 0.519, 95% CI = 0.344–0.805), Child‐Pugh class (B, *P *=* *0.0130, HR = 1.849, 95% CI = 1.145–2.875), serum DCP level at baseline (median level of ≥738 mAU/mL, *P *=* *0.0010, HR = 1.816, 95% CI = 1.271–2.601), and duration of treatment (median duration of ≥3.6 months, *P *<* *0.0001, HR = 0.285, 95% CI = 0.197–0.408) were independent risk factors for overall survival in patients with extrahepatic metastasis.

**Table 4:** Univariate and multivariate analyses of overall survival in patients with extrahepatic metastasis

Variable	Univariate analysis		Multivariate analysis	
	HR (95% CI)	*P*‐value	HR (95% CI)	*P*‐value
Age (≥72 years)	1.310 (0.836–2.057)	0.2379		
Gender (male)	0.533 (0.315–0.937)	0.0297	0.519 (0.344–0.805)	0.0040
Child‐Pugh class (B)	2.242 (1.156–4.113)	0.0182	1.849 (1.145–2.875)	0.0130
Tumor stage (BCLC‐C)	0.692 (0.197–4.406)	0.6419		
Initial dose (800 mg)	0.808 (0.493–1.302)	0.3857		
Daily average dosage (≥400 mg)	1.200 (0.762–1.909)	0.4322		
AFP (≥105 ng/mL)	0.969 (0.554–1.696)	0.9124		
AFP‐L3 (≥22%)	0.931 (0.559–1.541)	0.7838		
DCP (≥738 mAU/mL)	1.966 (1.252–3.120)	0.0032	1.816 (1.271–2.601)	0.0010
Duration of treatment (≥3.6 months)	0.268 (0.162–0.439)	<0.0001	0.285 (0.197–0.408)	<0.0001
Therapeutic effect (PD)	1.105 (0.729–1.692)	0.6386		

HR, hazard ratio; 95% CI, 95% confidence interval; BCLC, Barcelona Clinic Liver Cancer; AFP, alpha‐fetoprotein; DCP, des‐gamma‐carboxy prothrombin; PD, progressive disease.

## Discussion

Sorafenib, an oral multikinase inhibitor and a new molecularly targeted therapy for advanced HCC, has been shown to offer significant survival benefit with good tolerance by two randomized phase III placebo‐controlled trials 12, 15. Thus, it has become the standard treatment for advanced HCC. The present study prospectively assessed the safety and efficacy of sorafenib and identified the factors associated with survival in advanced HCC patients. The MST and PFS of patients receiving sorafenib in this study were 10.6 and 3.8 months, respectively. This MST was longer than what was observed in the Asia‐Pacific study (6.5 months) and comparable to the SHARP trial's result (10.7 months).

An exploratory multivariate analysis identified four baseline patient characteristics as prognostic indicators for overall survival, including gender, Child‐Pugh class, serum DCP level at baseline, and duration of treatment. However, dosage and therapeutic effect of sorafenib were not significant risk factors adversely affecting survival in this study. It is noteworthy that men treated with sorafenib had higher survival rates than women. Although the reason for such a result is unclear, physical constitution might be a possible contributor. Moreover, the median treatment duration was 3.6 months in men and 3.0 months in women, suggesting that women might not be able to tolerate sorafenib treatment as well as men. Previous studies reported that in HCC patients, high serum DCP levels were associated with vascular invasion, metastasis, and tumor recurrence 22. Furthermore, DCP was more useful as an HCC marker in larger tumors, which were more likely to be exposed to hypoxia during development. Thus, higher serum DCP levels might be indicative of a more advanced HCC state with reduced survival rates.

The overall disease control rate was 47% in this study, including 18 (6%) patients with PR and 127 (41%) with SD, whereas 134 (43%) patients had PD. Notably, the proportion of patients with PR in our study was higher than that of the SHARP trial (2%) and the Asia‐Pacific study (3.3%). However, the reason for such a result is unclear. Previous studies suggested that there could be racial differences in terms of gene mutations that might affect sorafenib treatment 23. Thus, Japanese patients with advanced HCC might be more sensitive to sorafenib than Western and other Asian patients. Further studies, with larger patient populations, are needed to investigate possible differences in the therapeutic effects of sorafenib.

Treatment‐related adverse events are an important issue affecting the continuation of sorafenib treatment. In this study, although the overall incidence of treatment‐related adverse events was high (86%), the observed events were primarily controlled with medical treatment and sorafenib dose reduction. Adverse events leading to discontinuation of treatment included liver dysfunction (14%), HFSR (2%), and diarrhea (2%), which are commonly associated with sorafenib 24, 25. However, in the SHARP trial, the overall incidence of treatment‐related adverse events was 80% in the sorafenib group, and the most frequent adverse events leading to discontinuation of sorafenib treatment were gastrointestinal events (6%), fatigue (5%), and liver dysfunction (5%) 12. HFSR is particularly well known as an early adverse event 26, 27, 28 associated with sorafenib therapy, and the severity of HFSR depends on treatment duration, dosage, and drug accumulation 29. Further effort spent toward the effective control of adverse events and management of sorafenib dosing, with a priority given to facilitating long‐term administration, will lead to the most effective therapy for HCC patients. Moreover, hepatic reserve is important for hepatic extraction and metabolism of sorafenib.

The prognosis of HCC patients with extrahepatic metastases is unsatisfactory 30, 31, but not well understood. In the present study, we assessed the prognosis of 312 consecutive HCC patients with or without extrahepatic metastases. We found that the most frequent metastatic sites were the lungs, bone, and lymph nodes. In addition, the overall survival and radiologic PFS time did not differ significantly between patients with and without extrahepatic metastasis. Multivariate analysis showed that independent risk factors for decreased overall survival in patients with extrahepatic metastasis were similar to those affecting all patients. Thus, the presence of extrahepatic metastasis might not affect the prognosis of advanced HCC patients treated with sorafenib. Moreover, previous studies reported that advanced HCC with extrahepatic metastasis often had poor prognosis. Therefore, these patients should be considered for sorafenib treatment.

In conclusion, our results indicated that the therapeutic effect of sorafenib was comparable in advanced HCC patients with or without extrahepatic metastasis. In addition, this study demonstrated that sorafenib should be administered for hepatic reserve and as long‐term treatment for advanced HCC patients.

## Conflict of Interest

None declared.
